# Optimal seismic retrofitting techniques for URM school buildings located in the southwestern Iberian peninsula

**DOI:** 10.1371/journal.pone.0223491

**Published:** 2019-10-16

**Authors:** María-Luisa Segovia-Verjel, María-Victoria Requena-García-Cruz, Enrique de-Justo-Moscardó, Antonio Morales-Esteban

**Affiliations:** Department of Building Structures and Geotechnical Engineering, University of Seville, Seville, Spain; Pablo de Olavide University, SPAIN

## Abstract

This paper aims to study different seismic retrofitting techniques to test the reduction of the seismic vulnerability of unreinforced masonry buildings. Three techniques have been considered in a case study: adding steel or carbon fibre reinforced polymer grids in the walls and steel encirclements in the openings. The performance-based method has been used to that purpose. Nonlinear static analyses have been performed to obtain the capacity and fragility curves, the performance point and the damage level states. Moreover, an analysis of the cost-benefit ratio has been carried out. Results have shown that the three techniques have produced considerable improvements. The addition of encirclements has reduced the deformation resulting in a slight increase of the structure’s stiffness. Adding steel grids has produced the maximum peak strength increase while adding polymer grids has produced the largest ultimate displacements. Adding encirclement has had the best cost-benefit ratio.

## Introduction

Throughout history, earthquakes have severely affected buildings causing numerous human casualties and economic losses [1]. In this context, buildings’ vulnerability is a key aspect to focus on to reduce these catastrophic effects. Generally, unreinforced masonry (URM) buildings present a worse seismic behaviour as compared to reinforced concrete buildings [2]. This is due to the low stiffness and strength of their components. Consequently, modern seismic codes include recommendations aimed to reduce their seismic vulnerability. However, a major part of the URM building stock has been constructed prior to these codes, thus considering unrestrictive requirements.

In the case of Spain, the seismic code that first introduced a seismic action value for Huelva (PGS-1) was published in 1969 [3]. Nevertheless, its compliance requirements were not as explicit as those established by the later seismic code published in 1994, the NCSE-94 [4]. This led to a lack of seismic considerations in the buildings’ design process prior to 1994 [5].

In this context, a project named PERSISTAH “*Projetos de Escolas Resilientes aos Sismos no Território do Algarve e de Huelva*” is under development [6]. The project aims to assess and reduce the seismic vulnerability of primary school buildings located in the Algarve-Huelva region (Portugal-Spain) [7]. In the PERSISTAH framework, a database of the region’s schools has been created. This database includes information regarding the constructive, structural and soil characteristics of the schools. The data has been obtained from original blueprints, in-site visits to the buildings and online questionnaires sent to schools. A total amount of 138 schools were identified in Huelva. Half of them were constructed with URM walls in the period from the 50s to the 80s.

In this project, school buildings have been selected for analysis owing to the especial vulnerability of their occupants (i.e., the children). Factors such as their capacity to evacuate the building in case of an earthquake, the low ratio between adult/child as well as the trauma that children can suffer after the event [8] make children as the least resilient part of the society [9]. Not only the occupants and the building’s configuration affect the vulnerability, but also the seismic hazard of the area. In this case, the Algarve-Huelva region is characterized by its proximity to the Azores-Gibraltar fault [10]. This area has been historically affected by some of the most catastrophic earthquakes suffered in the Iberian Peninsula. These earthquakes are characterized by large magnitudes (Mw≥6) and long return periods [11]. Despite the large magnitudes but precisely because of the long return periods, the population is unaware of the seismic hazard of the area. As a result, there are few studies on the seismic hazard of the Algarve-Huelva region [7]. Moreover, neither risk prevention nor seismic retrofitting policies have been established for buildings in this area. At present, the Eurocode-8 part-3 (EC8-3) [12] establishes that URM buildings can only be constructed in areas of low seismicity (*a*_g_<0.08g) (where *a*_g_ is the EC8 design ground acceleration). In the case of Huelva, all the municipalities located in the southern part of the province have higher values of *a*_gR_ and in case of the Algarve region the minimum value of *a*_gR_ is 0.15g. Therefore, the URM school buildings located in this area (in fact, the most populated) do not comply with the EC8-3 provisions. Consequently, the study of the seismic vulnerability of these buildings is prominent.

The seismic vulnerability of URM buildings can be reduced by an effective and efficient seismic retrofitting [13]. Thus, this paper aims to study the effect of different seismic retrofitting techniques to reduce the seismic vulnerability of an URM school. The analysis has been carried out considering a primary school building located in the Algarve-Huelva region. The building has been selected due to its suitable sizeand the seismic hazard of the area. In fact, this building was designed and built with no seismic consideration. Furthermore, this is representative of a considerable number of similar buildings in the area. Three different URM buildings retrofitting techniques have been considered in the analysis. The performance-based method has been used to obtain the strength increase and the damage level reduction produced by the addition of these techniques. Nonlinear static analyses have been performed to obtain the capacity and fragility curves, the performance point (PP) and the damage level states. The results have been compared with the original building seismic performance. Moreover, an analysis of the cost-benefit ratio has been carried out. The retrofitting techniques analysed are of low architectural impact and can be easily implemented.

### Retrofitting techniques for URM buildings

A varied amount of techniques for the seismic retrofitting of URM buildings have been analysed in the literature. The most common categories of retrofitting strategies used are (a) reinforcement of connections (wall to wall, wall to floor or wall to roof), (b) transformation of flexible floors into rigid diaphragms, (c) improvement of the out-of-plane behaviour through tied rods or ring beams, and (d) reinforcement of masonry panels. These techniques have been reported to be effective in numerous studies [14][15][16].

In the case of the Algarve-Huelva region, the major part of the URM school buildings do not present problems related to the connection between elements. Furthermore, they are composed of rigid diaphragms and have reinforced concrete ring beams on the top level of the walls. Consequently, this study is focused on the reinforcement of panels, which is the most suitable retrofitting technique to improve its behaviour. In addition, the techniques considered in this study do not modify the buildings’ configuration and are reversible.

The materials more widely used in the reinforcement of masonry panels are: (1) steel used in profiles or rebar; (2) reinforced polymers, such as carbon or glass fibre used as bands; (3) cement based materials used as mortar renders reinforced with glass or textile fibres; (4) cement grouting or (5) reinforced concrete. This last material is used to create new frames or to reinforce the walls’ core as proposed by [17]. Two materials have been selected for the analysis carried out in the present study. Firstly, the steel as profiles or grids, owing to its low cost and easy implementation. Secondly, the Carbon Fibre Reinforced Polymers (CFRP), due to its high efficiency and the considerable improvement of the strength and the dissipative capacity of the walls.

Steel has been widely used to retrofit URM walls as grids anchored to panels and covered by cement mortar. According to the grid’s type and the anchorage’s technique, different solutions can be found. The first solution is based on the use of the ferrocement. In this case, the grids are composed of welded wire meshes and covered with micro concrete mortar [18]. A second solution implements a reinforced plaster with grids of intermediate diameters (4 to 6 mm) anchored to both wall’s faces [19]. A third solution is based on the use of shotcrete. In this case, higher diameter grids (6 to 13 mm) are used, and the concrete is projected on the grid [20]. A different solution based on the aforementioned techniques is the Cam System. In this case, steel plates are used instead of grids. They are added on both wall’s faces and are anchored between them to create a 3D anchorage system of the wall. These plates and anchorages are first prestressed to comprise the panel [21]. Studies implementing this solution concluded that it was rather effective [22] due to the considerable increase in the resistance and the ductility of the structure.

These techniques have been analysed in different studies to test their effectiveness. In [19], the global seismic response of reinforced plaster grids was tested. The authors compared the maximum displacements, the inter-storey drifts and the maximum stresses. Results showed that the application of reinforced plaster proved to be beneficial in terms of the improvement of the seismic response, resulting in an overall stress minimization. In [15], several models that added reinforced plaster were analysed. In this case, the authors used reinforced plaster made of a steel welded mesh of 0.5 mm thickness and 10 mm spaced, covered by a 3 cm mortar layer. They concluded that technique was able to fulfil the ultimate limit states’ requirements of current seismic codes in two case study URM buildings.

CFRP has also become one of the most used materials in the seismic retrofitting of URM buildings, owing to several advantages: low weight, mechanical properties, lack of corrosion and application feasibility [23]. In the case of CFRP the major part of the studies is focused on testing its diagonal compression resistance by applying cyclic loads to assess the in-plane behaviour of the reinforced panels. In [24], the authors tested wide fibre bands in vertical, horizontal and diagonal. Also, in [25], the bands were narrower and also rebars were embedded in grooves. The addition of these solutions resulted in a considerable improvement in the resistant and dissipative capacity of the wall. However, as concluded by [26][27][28], this technique presents an important weakness, namely the lack of adhesion between the bands and the masonry panel. In [29], a four-story residential building was strengthened with CFRP bands of different thicknesses and configurations. The analysis concluded that the CFRP bands presented a good capability of preventing structural collapse with minor local damage.

In URM buildings, the presence of openings in the walls decreases the in-plane seismic capacity. In [30], the authors concluded that the openings significantly affect the seismic performance of the wall, producing a concentration of shear force and drift demands in some parts of the wall. Similar conclusions were obtained in [31] by experimentally analysing the effects of windows and doors openings in walls. They found that the opening ratio is a key parameter on the shear strength of walls. Moreover, the results showed consistent damage patterns usually concentrated in the wall piers. This drawback has been also observed in the inspections of school buildings after strong earthquakes [32]. In the case of the 2002 Molise (Italy) earthquake, URM panels with higher opening ratio presented deeper cracks in the walls. Therefore, the presence of high ratios of openings considerably increases the seismic vulnerability of URM buildings.

In this context, a recent study experimentally analysed a new technique to strengthen the masonry walls [33], consisting in a steel encirclement installed in the openings. The wall specimen was cyclically tested to failure. The results showed that the encirclements led to a significant increase in strength and in-plane deformation capacity, as well as in cumulative dissipated energy at collapse. Comparing a retrofitted wall to a solid masonry wall (without openings), the results were similar, especially in terms of peak strength. Nevertheless, despite the fact that the openings are the prominent weakness of URM buildings, there is a lack of studies analysing their reinforcement.

In the present work, three different techniques of wall reinforcement, including the steel encirclements technique, have been assessed and compared by means of the pushover analysis of a case study building under different retrofitting conditions. The detailed analysis of results in terms of capacity and fragility allowed obtaining relevant conclusions regarding the impact in the seismic behaviour of the different retrofitting options and parameters.

## Method

### Case study

In Huelva, 138 primary schools have been identified within the PERSISTAH project. Half of them were constructed with URM walls during the 50s and 80s. The building selected as case study is representative of these buildings since they were constructed during the same period, sharing the same constructive and structural techniques and codes. Moreover, the configuration, the built area and the distribution are similar.

The building selected is part of a school’s set named as “*Los Llanos*” located in Almonte. This town is situated in the southeastern Huelva, which is the area of highest seismic hazard of the province. Moreover, this is one of the most populated towns of the area.

**Fig 1:**
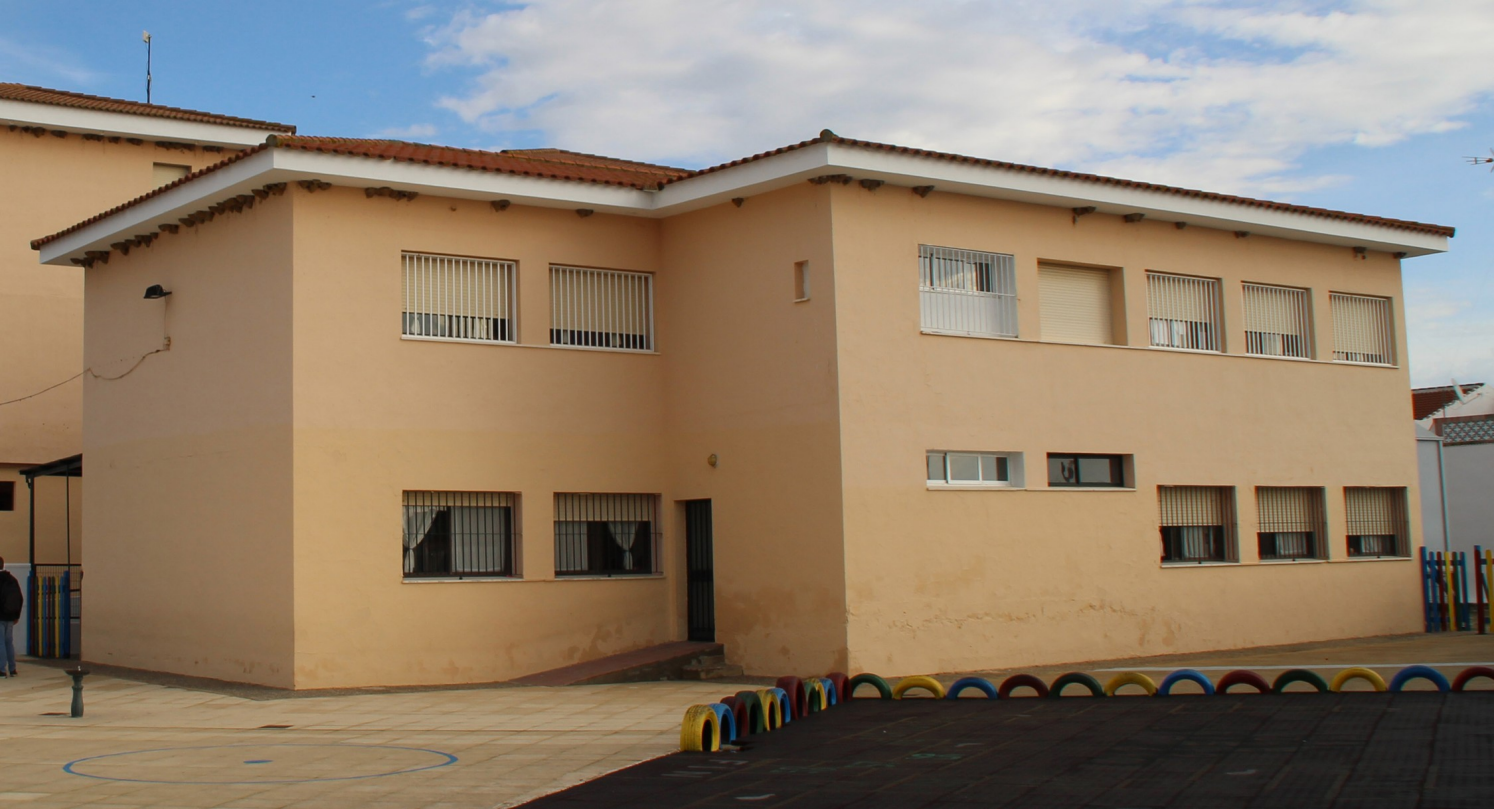
Façade of the building selected as case study (Authors’ ownership).

The school was constructed in 1986 and is used by pre-school children. This is a two-storey building characterized by a regular and compact geometry generated by the intersection of two volumes (Fig 1).

**Fig 2:**
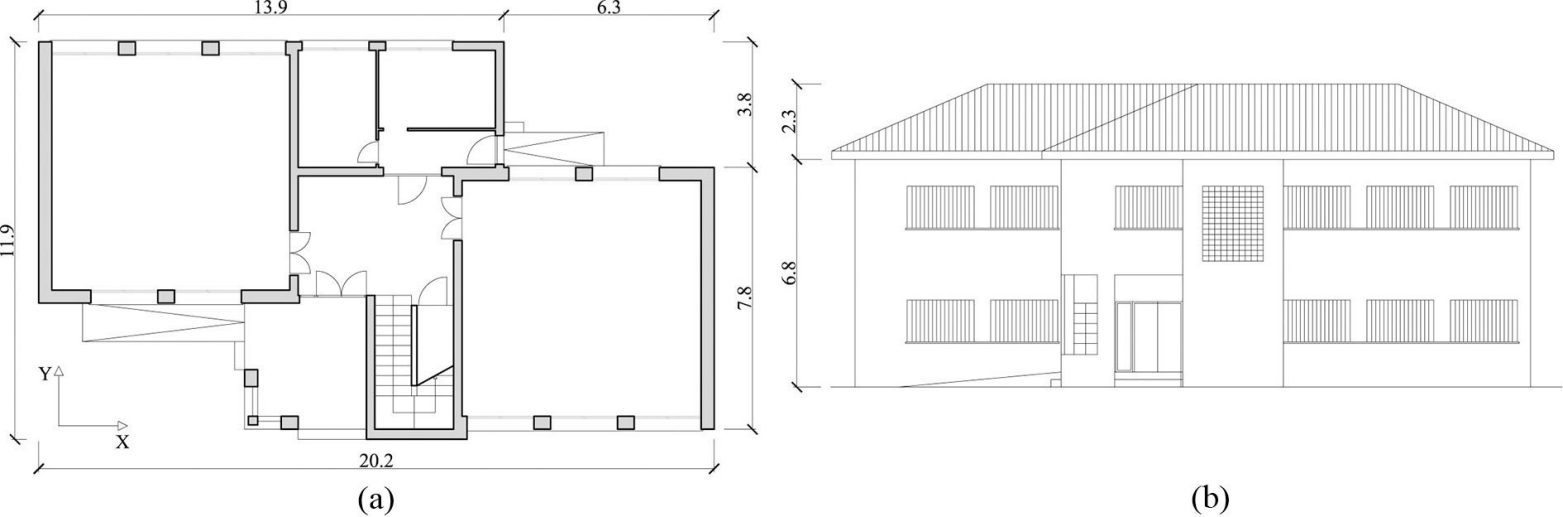
Distribution of the ground floor (a) and main façade (b) of the case study school.

The building was constructed with URM walls homogenially distributed in each orthogonal direction (Fig 2A). The total wall length in the X and Y direction is similar. The walls are 25 cm thick and are composed of clay brick and cement mortar, with 25x35 reinforced concrete ring beams placed on the top to reinforce the walls together. The foundation is a concrete beam on band footings. The building has a sloping roof with tiles. The most important seismic weakness of the building is the heterogenean distribution of the openings. The opening ratio is much higher in the X direction (Fig 2B), reaching up to 25%, while, the walls in the Y direction are blind walls.

The characterization of the masonry walls in existing buildings is complex. In this study, the mechanical characteristics have been obtained from the building codes and the project’s blueprints. The characteristic compressive strength (*f*_*k*_) has been determined through the empirical formula (Eq 1) established in the Eurocode-6 (EC6) [34].

$$f_{k}=\text{K}f_{b}^{\alpha }f_{m}^{\beta }$$ (1)

Where:

*K* is a constant that depends on the brick and the mortar type. It can be obtained from Table 3.3 of the EC6.
*f*_*b*_ is the compressive strength of masonry units.
*f*_*m*_ is the mortar resistance.

The value of *f*_*b*_ has been determined according to the Spanish building code applicable at the year of construction (MV-201) [35], which is 15 N/mm^2^. The *f*_*m*_ has been obtained from the technical data specified in the blueprints, being 4 N/mm^2^. The masonry elastic modulus (*E*) established in the EC6 is excessive for buildings constructed during the 1970 and the 1980s according to [36]. Hence, the authors of this work recommended the use of the French UIC Code 778–3 [37]. The procedure established in this code is more realistic and depends on the elastic modulus of the brick (*E*_*b*_) (Eq 2).

$$E=0.35E_{b}$$ (2)

In this French code, the *E*_*b*_ for a medium-hard brick was estimated as 10,000 MPa. Moreover, this value has been compared with the Italian NTC 2008 code [38] values. This latter code established a similar value for a masonry wall with similar compression resistance. In Table 1, the mechanical properties adopted for the brick masonry walls are listed.

**Table 1:** Mechanical properties adopted for the brick masonry walls.

Structural parameter	Value
Compressive strength (*f*_*k*_)	5 MPa
Shear strength (*t*_*0*_)	0.24 MPa
Young´s modulus (*E*)	3,500 MPa
Shear modulus (*G*)	875 MPa
Weight density (*W*)	15 kN/m^3^

The loads used in the analysis have been obtained from the original blueprints and the Spanish code CTE-DB-AE [39]. The dead load assigned to the ground and first floor has been 5.3 kN/m^2^. This load includes the weight of the concrete slab, the flooring material and the partition walls. The load applied in the roof floor has been 6.3 kN/m^2^. It includes the weight of the floor slabs and the construction elements of the gable roof. The live load assigned to the school floors has been 3 kN/m^2^ corresponding to public use buildings. In the roof floor, the live load determined has been 1 kN/m^2^ for maintenance only.

### Seismic performance-based assessment

The seismic performance analyses of URM buildings can be carried out by means of the seismic performance-based method [40]. There are several methods for the seismic performance analysis of buildings [41][42][43]. In this work, the N2-method [44] has been used, which is the method established in the EC8. It considers the capacity curve of the building and the inelastic demand spectrum. The method intersects both curves to determine the performance point (PP) of the building, which represents the expected displacement of the building for a given seismic action [45].

Masonry walls present a nonlinear behaviour owing to the low values of tensile endurance. Therefore, nonlinear static (pushover) analyses have been carried out to determine the building’s capacity curve in the X and Y directions [46]. These analyses apply an incremental horizontal load to reach the building’s collapse. Two load patterns have been considered according to the EC8: one proportional to the masses and another proportional to the first mode of vibration. The capacity curve obtained represents the basal shear force versus the displacement of the control node. This node must be located in the superior floor and presents the highest displacement value.

The inelastic demand spectrum has been defined according to the EC8 elastic response spectrum procedure. The seismic action has been obtained from the 2012 Spanish update of the ground acceleration values [47]. In Almonte, a value of PGA of 0.1g was determined. This acceleration must be multiplied by the importance factor (*γ*_*I*_) to obtain *a*_*g*_. In this case, schools are included in the category of especially important buildings. Therefore, a *γ*_*I*_-factor of 1.3 has been selected. The soil type has been determined according to a nearby geotechnical study carried out in 2014. Different Dynamic Probing Super Heavy (DPSH) tests were performed and the soil type was defined as C. This was characterised by the presence of silt-sand with medium-low compactness. Therefore, the soil coefficient value is 1.2.

### Building’s modelling

Nonlinear static analyses have been carried out by means of the TREMURI software [48], which implements the equivalent frame model approach (EFM). The EFM approach has been widely used for the seismic assessment of URM buildings due to its relative simplicity and calculation speed as compared to Continuous Constitutive Laws Model (CCLM) [49]. Moreover, this method has shown sufficient accuracy in the evaluation of the seismic performance of buildings using only a few mechanical parameters [50]. The masonry walls are divided into macroscopic structural elements such as piers and spandrels in which the strain is concentred [51]. Each element is defined by nonlinear constitutive laws in terms of forces and displacements and by some failure and drift limit criteria [52]. This approach requires a limit number of degrees of freedom and allows performing 3D analyses [53]. Moreover, it enables to analyse the nonlinear mechanical behaviour of walls, the expected damage and the failure types [54].

Two load patterns have been considered: one proportional to the masses and one proportional to the first vibration mode. Besides, the possibility of an accidental eccentricity of 5% has been considered for each load pattern and direction. Therefore, a total amount of 24 calculations has been carried out for each calculation.

**Fig 3:**
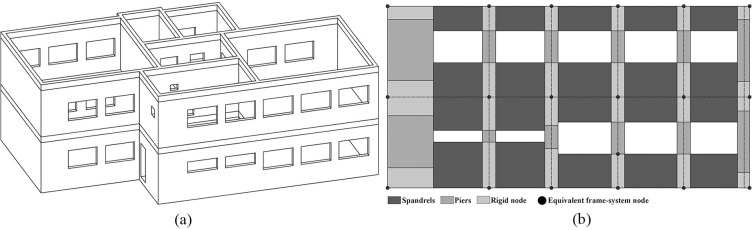
**Modelling of the 3D building (a) and macroelements of the south wall (b)(Authors’ ownership)**.

The EFM approach in pushover analyses has been widely applied to assess the efficiency of different seismic retrofitting techniques such as in [14][15]. The model of the school is shown in Fig 3A. In Fig 3B, the macroelements of the south wall are depicted as well as the distribution of the EFM system.

### Damage assessment

Once obtained the capacity curves, the fragility curves have been determined according to the method described in [55]. These curves determine the probability of reaching or exceeding a specific damage state for a given structural response and seismic action. The fragility curves have been obtained through the HAZUS [56] methodology, which is the method established in the PERSISTAH software. The required parameters have been obtained from the study carried out within the PERSITAH framework. The fragility curves follow a lognormal probability distribution and are constructed according to the spectral displacement [57]. Using the fragility curves and the PP’s spectral displacement, a mean damage index (DI) has been obtained by means of the fragility curves and the PP’s spectral displacement (Eq 3) [58]. The DI represents the state of damage of higher probability of occurrence for a given seismic scenario [59]. The damage states represent the effects that an earthquake can cause on buildings. The damage states considered in this study have been no-damage, slight damage, moderate damage, severe damage and collapse. A comparison of the DI reduction resulted from each retrofitting technique addition has been carried out.

$$DI=\sum_{i=0}^{n}i\;\times P\left(d_{s_{i}}\right)$$ (3)

Where:

*n* is the number of damage states. In this case, five: no-damage (0), slight damage (1), moderate damage (2), severe damage (3) and collapse (4).
*P(d*_*si*_*)* is the probability that a damage state *i* will occur.

### Retrofitting solutions

Three different retrofitting techniques have been considered with the aim of improving the seismic performance of the school building. All are non-invasive techniques and present reduced construction times. Two of them have been applied to the exterior face of the walls while the third has been implemented in the wall openings. In Table 2, the mechanical characteristics of the retrofitting materials are listed.

**Table 2:** Mechanical properties of the retrofitting materials considered.

Technique	Steel bars grid	CFRP grid	Steel framing
	(Steel B-500S)	(Fibre reinforced bands)	(Steel S275JR)
Elastic limit (*f*_*y*_)	500 MPa	-	275 MPa
Tensile strength (*f*_*u*_)	550 MPa	2,000 MPa	410 MPa
Modulus of elasticity (*E*)	200,000 MPa	170,000 MPa	210,000 MPa
Elongation at rupture	-	1.3%	-

**Fig 4:**
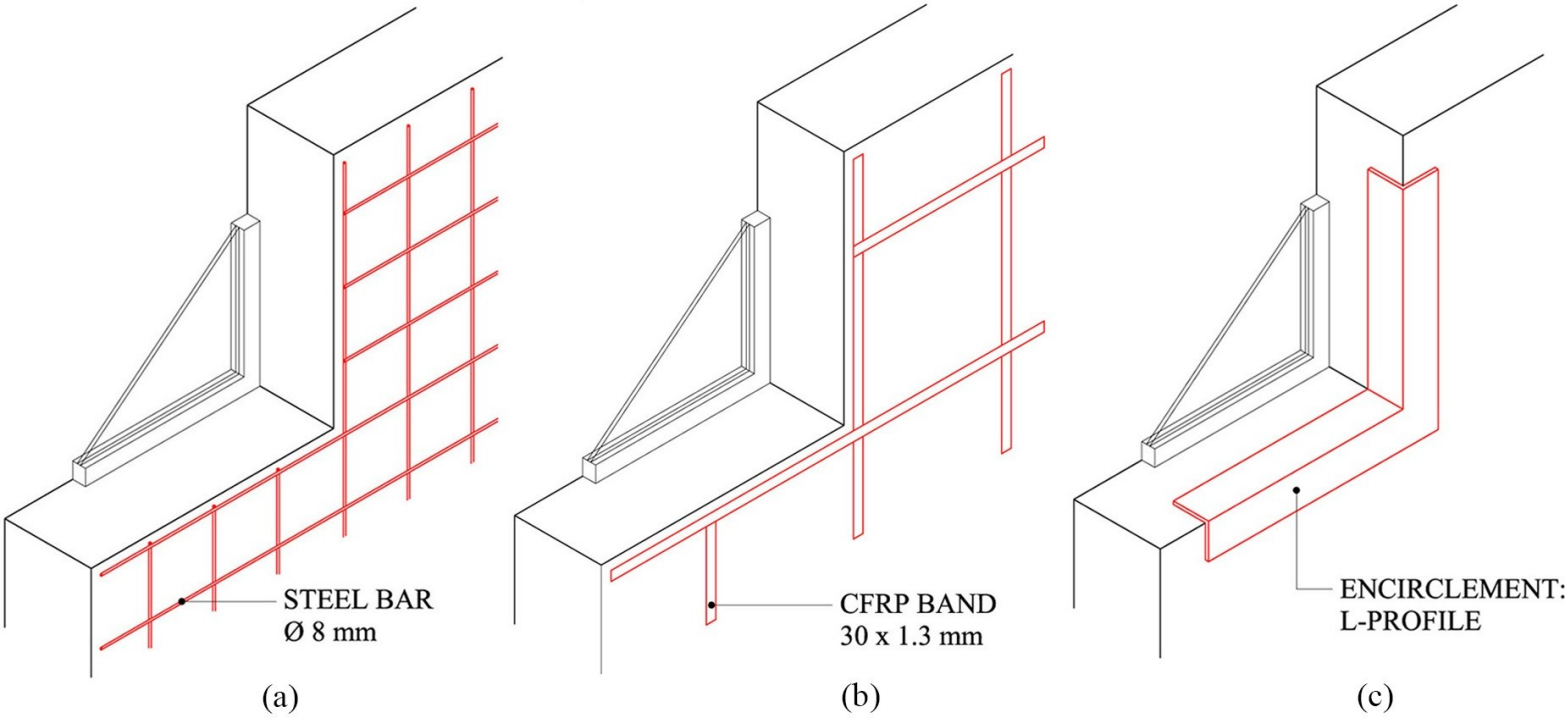
Retrofitting techniques: steel grid (a), CFRP grid (b) and encirclements (c).

The first technique is based on the addition of steel grids in the exterior faces of the walls as shown in Fig 4A. The material used has been steel B500S. The diameter of the rebars has been 8 mm and the bar spacing has varied from 20, 40, 60 to 80 cm. Previously to the addition of the grids, the paint must be removed. Then, an acrylic resin bonding layer must be applied between the existent and the new mortar renders. Next, the grids are mechanically anchored to the walls. Finally, a cement render and painting layer must be applied. The four cases studied have been named as S.G. Ø8-20, S.G. Ø8-40, S.G. Ø8-60 and S.G. Ø8-80, respectively.

The second technique analysed has been the addition of CFRP grids in the walls as shown in Fig 4B. In this case, bands of 30 mm width and 1.3 mm thick have been implemented in vertical and horizontal position. The band spacing has varied from 50, 100 to 150 cm. The construction process is similar to the first solution procedure. However, in this case, the mortar must be removed before adding the bands. The bands are bonded with a slender epoxy resin layer. The three cases studied have been named as CFRP-50, CFRP-100 and CFRP-150, respectively.

The third technique is the encirclement of the openings, carried out by adding L-shaped steel profiles. They have been mechanically anchored in the outer edge of the window frame, as shown in Fig 4C. Despite the fact that the L-profile is visible from the outside, the architectural impact is low. Moreover, this technique can be easily implemented. During the construction process, the windowsills are first removed and then placed back again. The four cases studied have been named as E. L50.5, E. L80.10, E. L100.10 y E. L120.12, each implementing a different L-shaped profile: L50.5, L80.10, L100.10 and L120.12, respectively.

The modelling of these three types of reinforcements has been carried out in TREMURI. The software enables to define a steel or CFRP grid in the walls’ properties definition. The effects of the addition of these retrofitting techniques are considered by improving automatically the mechanical properties of the walls. In the case of the retrofitting of openings, a steel or reinforced concrete encirclement can be added in the openings’ definition. This configuration is then incorporated to the definition of the mesh of each wall.

### Cost/benefit analysis

Similarly to [19], an analysis of the cost/benefit ratio has been carried out. The construction costs of each solution have been measured, using a Spanish construction cost database [60]. In case of the wall reinforcements, the cost of adding the steel and CFRP grids has been obtained per linear meter and only for the exterior side of the wall. In case of the encirclements, the cost is the sum of the costs of adding the steel profiles in each opening. A cost index has been calculated as the normalized ratio between the analysed solution and the most expensive one. After obtaining the cost, the benefit has been assessed considering the reduction of the DI resulted from the addition of the retrofitting technique. It has been calculated as the normalized ratio between the DI of the considered solution and the DI of the original building (without retrofitting).

## Results

In this section, the results of the analysis, in terms of performance, damage level and construction costs of the original and retrofitted building, are presented.

### Reference building

**Fig 5:**
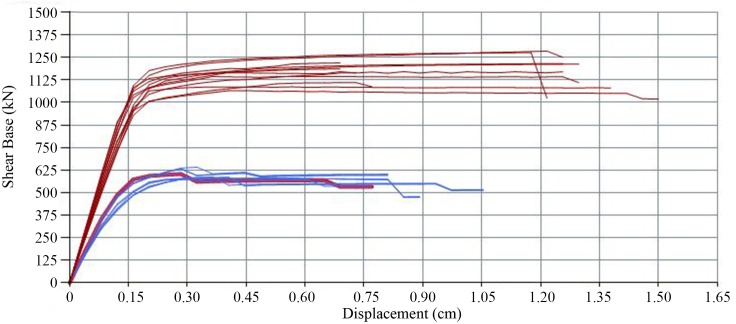
Reference building capacity curves for the ±X and ±Y direction considering two load patterns and positive, negative or null eccentricity.

Fig 5 shows the results of the pushover analysis of the original building. A total of 24 capacity curves for the +X, -X, +Y and–Y directions of the building have been obtained, corresponding to the different hypotheses considered (see section 3.3). Then, the performance points have been determined by means of the N2 method. In the X direction, the displacement of the most unfavourable hypothesis has been 0.77 cm and the basal shear force 581 kN. In the Y direction, the displacement has been 0.11 cm and the basal shear force 829 kN.

**Fig 6:**
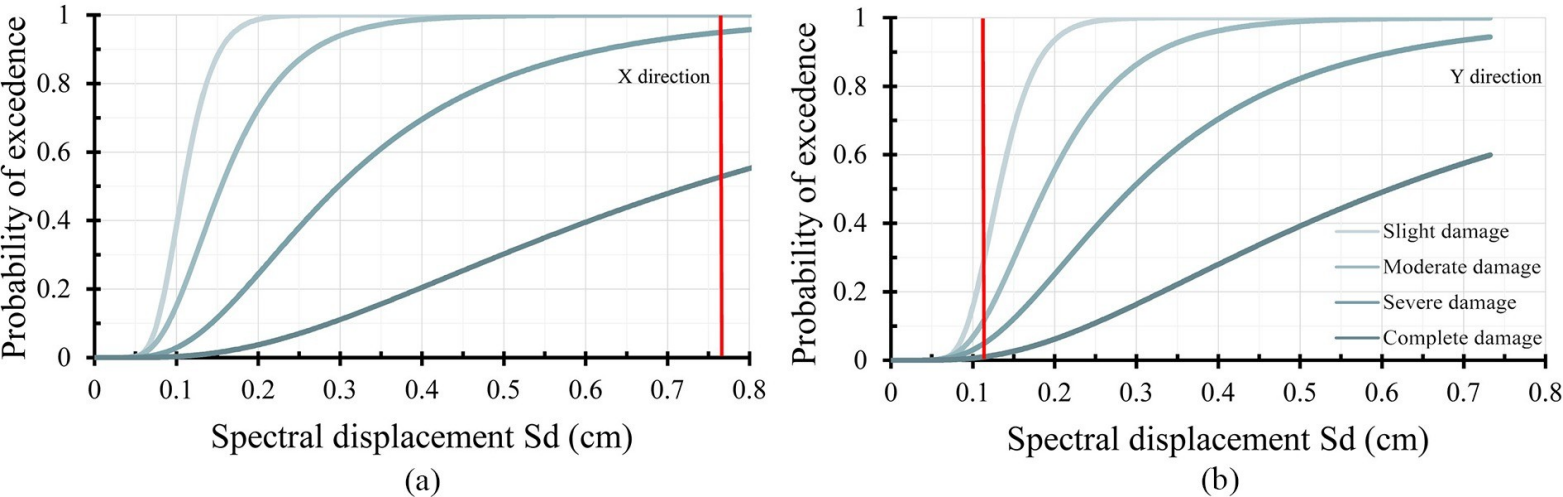
Reference building fragility curves in the X (a) and Y (b) direction, highlighting in red the PP displacement.

Once the capacity curves have been determined, the fragility curves have been obtained. Fig 6 shows the fragility curves corresponding to the reference building and the PP displacement in the X and Y direction.

The results show considerable differences regarding the building strength capacity in the X and Y direction. In X, the capacity is only half as much as strength in Y direction. Regarding the PP, the difference is more considerable: the displacement in X is 7 times greater than in Y. Consequently, the probability of damage is much greater in the X direction. In particular, higher percentages of severe and complete damage have been obtained in the X direction. Contrariwise, in the Y direction the probable damage level is minimum. These different results were expected owing to the higher ratio of openings in the X direction. Therefore, the analysis of the improvement provided by the different retrofitting schemes will be focused in the X direction, which is the one presenting a higher vulnerability.

### Reinforced building

**Fig 7:**
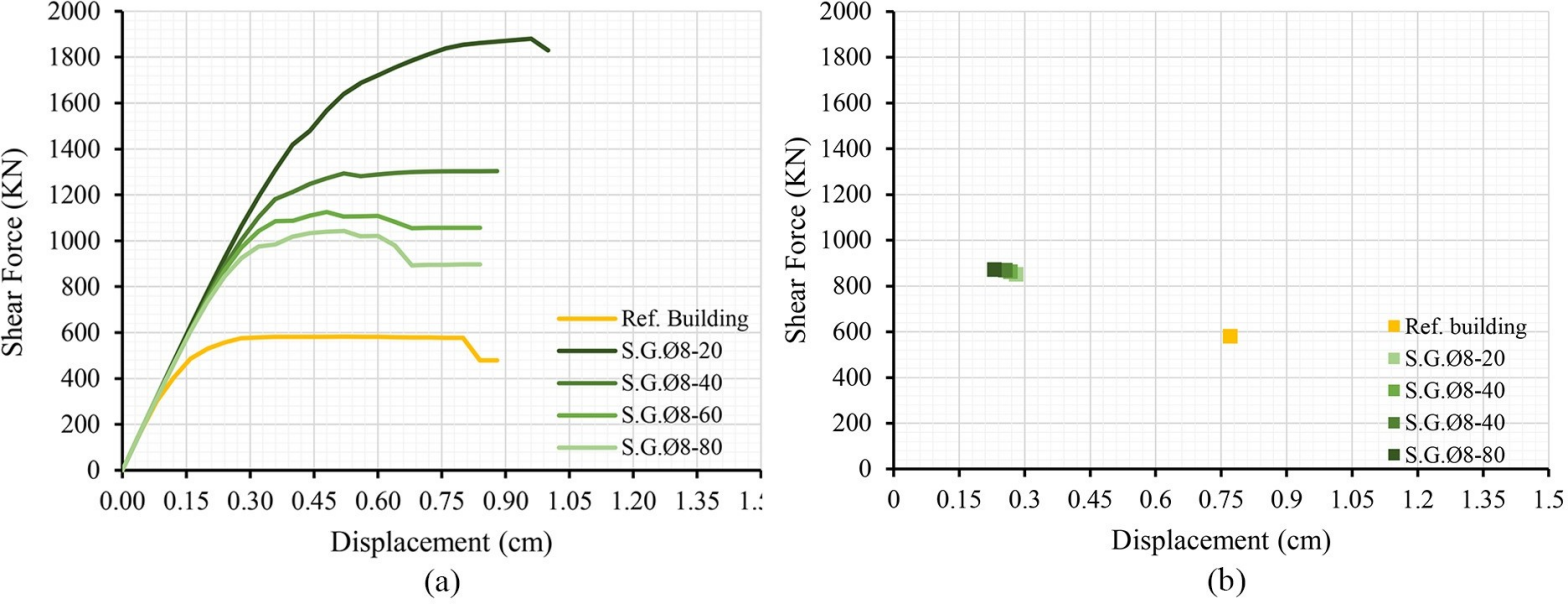
Capacity curves (a) and performance points (b) of the models adding steel grids varying the bar spacing.

**Fig 8:**
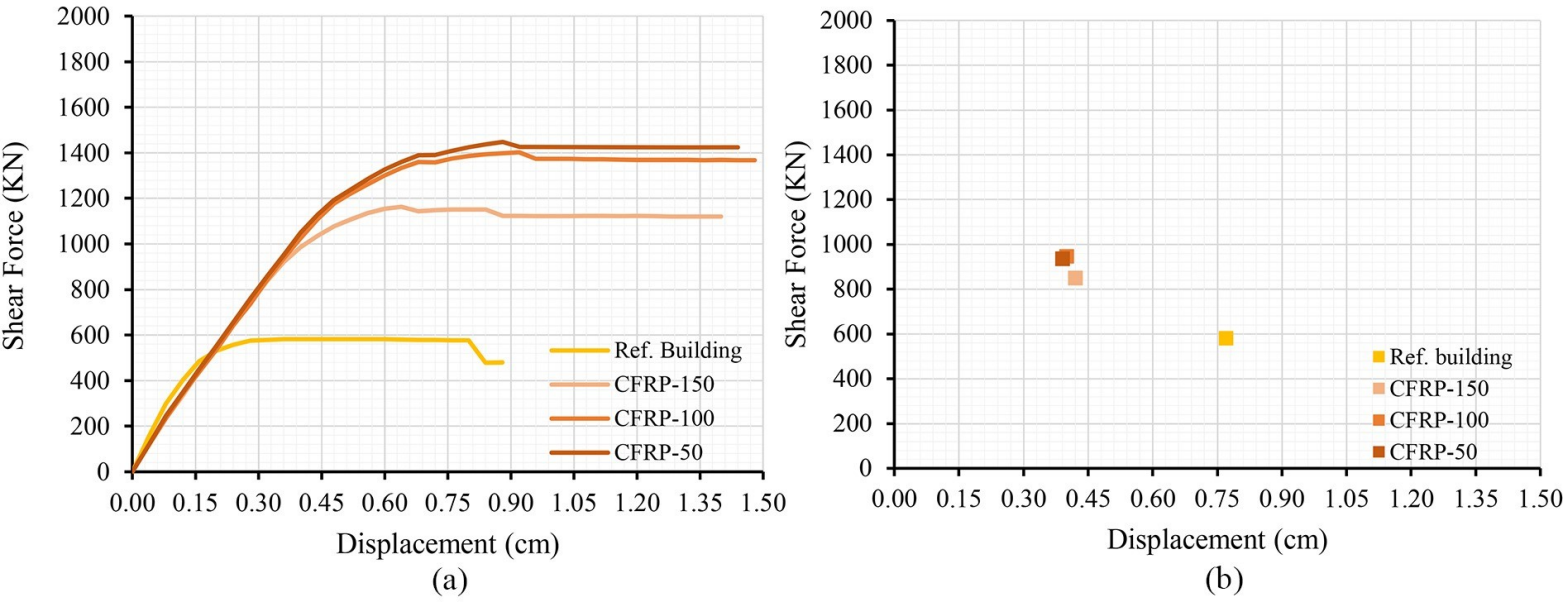
Capacity curves (a) and performance points (b) of the models adding CFRP bands varying the band spacing.

**Fig 9:**
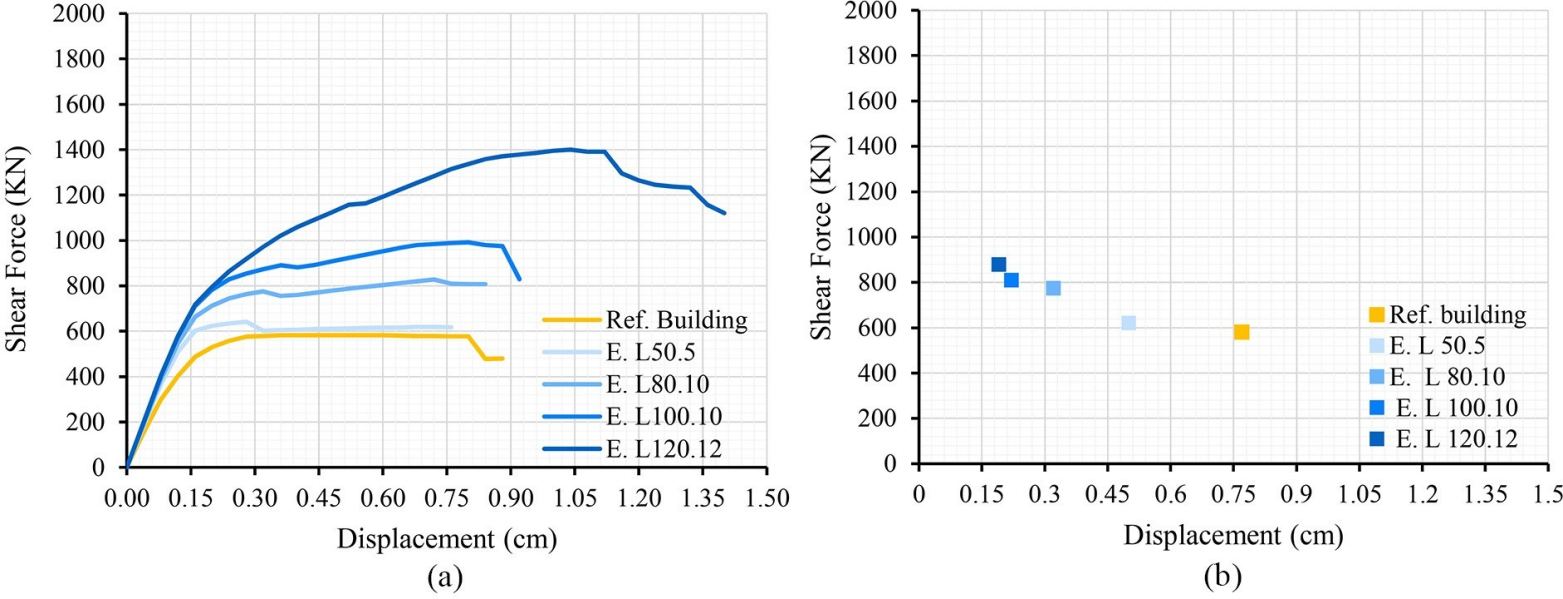
Capacity curves (a) and performance points (b) of the models adding steel profiles in the openings varying the profile size.

The results of the addition of the retrofitting techniques are shown below; in terms of capacity curves and performance point (Figs 7, 8 and 9). The most unfavourable curve has been selected in each case, considering two load patterns and a 5% eccentricity. The curve of the reference building has been included in order to compare it with the curves obtained.

**Fig 10:**
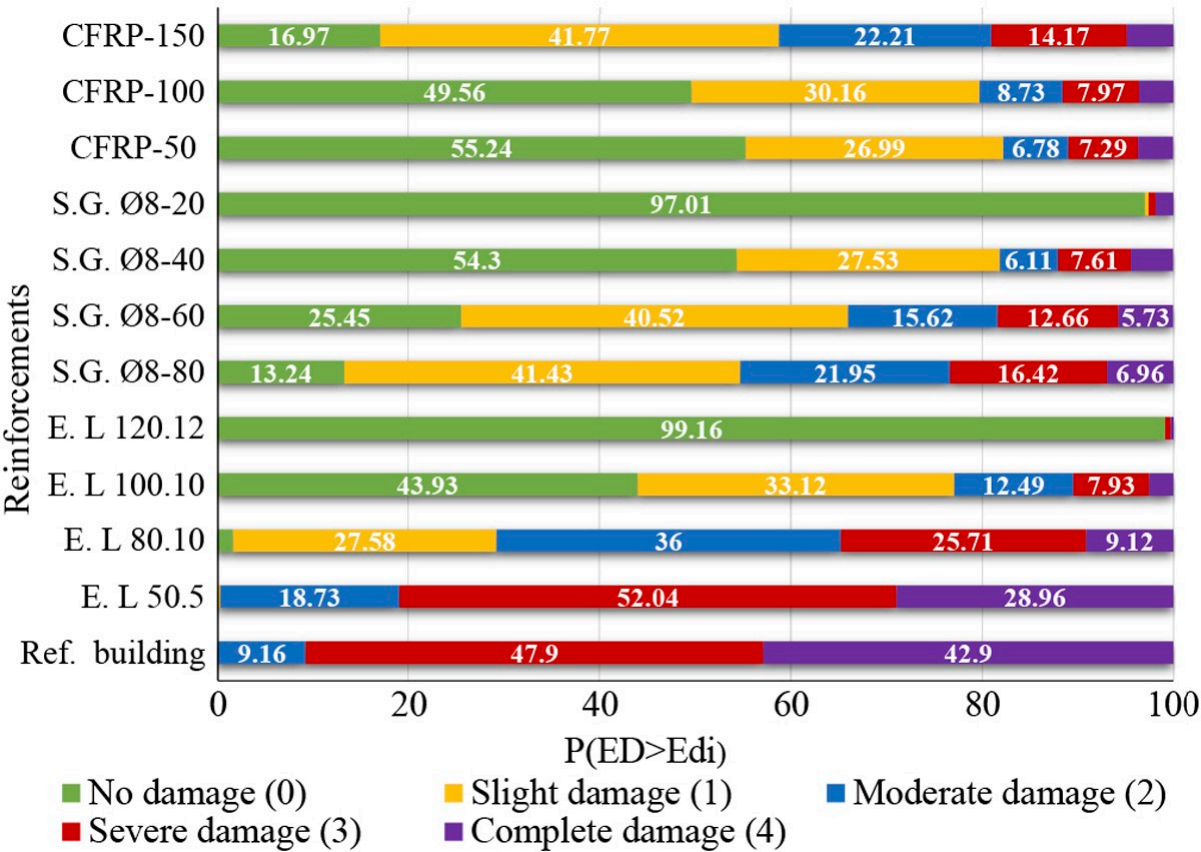
Damage states probabilities.

**Fig 11:**
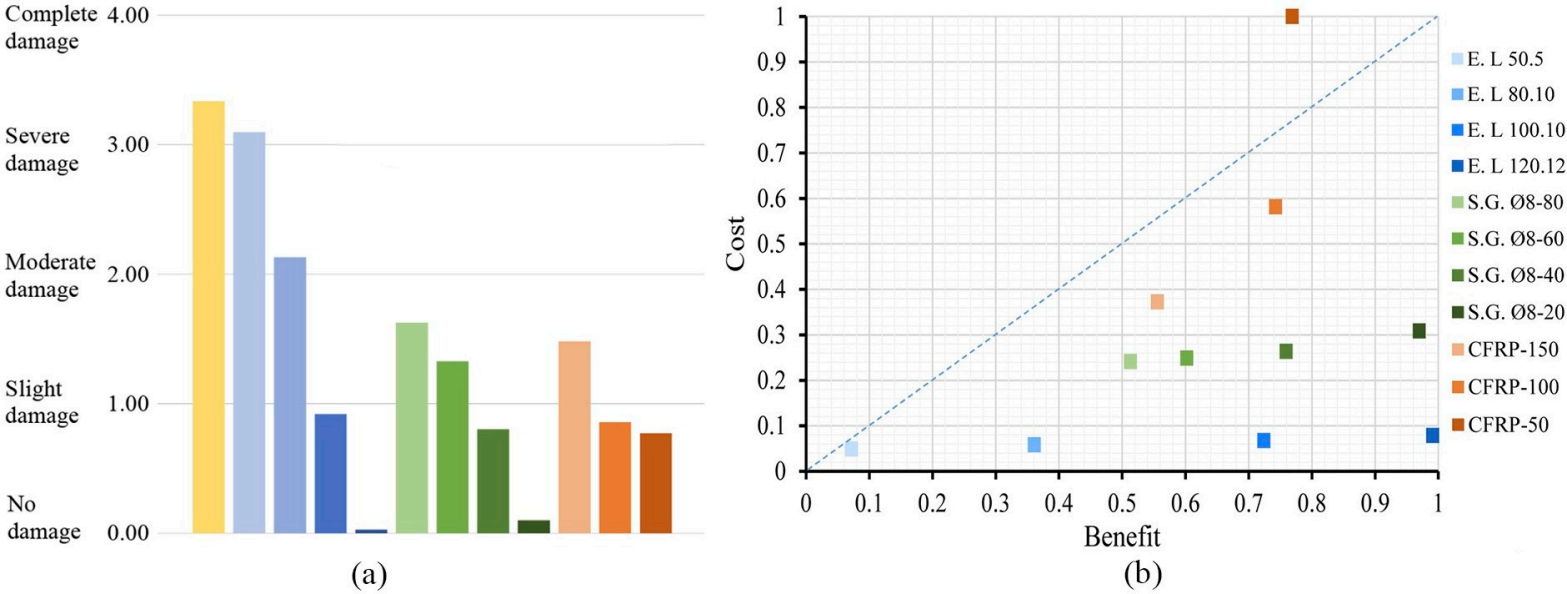
DI for each retrofitting solution (a) and cost-benefit index (b).

To assess the damage level for each retrofitting solution, the damage states probabilities have been obtained from the fragility curves and the PP’s displacements (Fig 10). In Fig 11A, the DI obtained for each retrofitting solution is shown. Finally, in Fig 11B, the results of the cost-benefit analysis have been plotted for each retrofitting solution.

## Analysis of the results

### Performance assessment

The results show that all retrofitting solutions have enhanced the seismic performance of the original school. The resistant capacity of the building has been increased whereas the PP displacement has been notably reduced.

Adding a steel grid spaced 20 cm (S.G. Ø8-20) has produced a maximum peak strength three times higher than the original capacity. Grids with higher rebar spacings (40, 60 and 80 cm) have increased the original capacity by 200% approximately. As for the PP displacement, values ranging from 0.2 to 0.3 cm have been obtained, which constitutes a significant reduction from the original PP displacement value (0.77 cm). On the other hand, varying the rebar spacing has resulted in no significant change in the values of the PP displacement. This is due to the fact that all capacity curves are similar for small displacements. The maximum capacity increase has been obtained for higher displacements ranging from 0.5 to 1 cm.

Concerning the CFRP bands, the resistant capacity of the building has increased from 100% to 160%, in the case of the CFRP-100 and CFRP-150, respectively. However, the improvement has not been noticeable when decreasing the band spacing from 100 to 50 cm (CFRP-100 and CFRP-50). A similar analysis was reported in [27], where the authors tested different walls retrofitted with different types of CFRP and concluded that adding fibres of better quality did not produced better results. Moreover, they concluded that the optimal retrofitting solution should determine a critical resistant value of the fibre according to the mechanical properties of thewall. In this study, no differences can be observed regarding the PP displacements. In all cases, the reduction of the 50% of the original displacement has been obtained.

The addition of encirclements has produced a different tendency. In this case, clear differences can be observed in the resistance increase generated by the four solutions. The solution with a L50.5 profile has not produced a significant enhancement. The L80.10, L100.10 and L120.12 profiles have produced an enhacement of the capacity in a 30%, 70% and 160%, respectively. These differences between profiles have been noticeable since the inicial phases of the calculations. Consequently, the values obtained for the PP have differed considerably. In fact, this variation has ranged between the 30% for the L50.5 profile and the 75% in the case of the L120.12 profile. Regarding the structural behaviour, in Fig 9A it can be observed that a stiffness increase in the initial deformation has been produced. This is due to the fact that openings can maintain the shape owing to the profiles addition. Therefore, the deformation is then initiated when higher loads are applied.

### Damage assessment

The results regarding the probability of reaching a damage state are useful to compare the effectiveness of the different retrofitting techniques. Moreover, it can be possible to obtain the quantity of retroffitting material needed to reach a notable enhancement of the building behaviour. Particularly, the addition of L50.5 encirclement has been the only solution that has not produced a significative reduction. Contrariwise, the addition of steel grids spaced 20 cm (S.G. Ø8-20) and L120.12 encirclements has produced the most important reduction in the damage levels. Moreover, they have reached values near the no-damage state. Adding L80.10 encirclements has not been considered safe enough since the DI obtained has been moderate damage. The rest of the solutions could be considered appropiate since the DI is lower than the moderate damage, close to slight damage.

In the case of the steel grids, all options have produced DI near the slight damage state, except the S.G. Ø8-20. Similarly, for the CFRP reinforcement, the DI is close to slight damage for the three cases, despite the fact that the band spacing has been decreased by 100% and 200%. Therefore, as previously mentioned, the determination of the most suitable band spacing value is crucial to achieve the optimal solution in order not to generate additional costs.

### Cost assessment

The cost analysis (Fig 11B) has revealed considerable differences in each construction costs’ solution. The addition of CFRP bands has resulted the most expensive technique. However, the encirclements have been the least expensive solution for all profile options considered. In the case of the steel grids and the encirclements, varying the amount of material does not impact significantly in the construction cost. This is due to the fact that the costs are mainly based on the workmanships costs, and not in the material quantity. Contrariwise, in the case of CFRP, the cost is much more dependent on the quantity of material added. The most expensive solution has not reached the maximum effectiveness, being only the 30% of the most effective solution. Overall, the addition of L120.12 encirclements has been proved to have the most optimal cost-benefit ratio.

## Conclusions

The PERSISTAH project aims to assess and reduce the seismic vulnerability of primary school buildings located in the Algarve-Huelva region, which can be achieved by an effective and efficient seismic retrofitting of the buildings. This paper has analysed and compared three different seismic retrofitting techniques to test the reduction of the seismic vulnerability of URM buildings. The analysis has been carried out in a primary school building located in the Algarve-Huelva region.

The three retrofitting techniques considered in the analysis have been: applying steel grids and CFRP bands in the walls and encirclements in the openings. Different bar and band spacing have been tested. In the case of the encirclements, the size of the profiles used has been modified. The performance-based method has been used to obtain the resistance increase and the damage level reduction produced by the addition of these techniques. Nonlinear static analyses have been performed to obtain the capacity and fragility curves, the performance point (PP) and the damage level states.

The results have shown that the retrofitting solutions have enhanced the seismic performance of the original school. The addition of encirclements has reduced the deformation, resulting in an increase of the structure’s stiffness. Adding steel grids has produced the maximum peak strength increase. Nevertheless, adding encirclements has resulted in the maximum reduction of the PP displacements. This is due to the moderate seismic demand of the region. Therefore, in these cases, the increase of the capacity has not been leveraged as the increase of the stiffness produced by the encirclements.

The damage level assessment has allowed to conclude that, in the case of the encirclements, noticeable different values of DI has been obtained and the size of the profile is crucial to obtain a required effectiveness. On the contrary, adding steel and CFRP grids has produced more homogenous results of the DI regardless of the bar or band spacing, especially in the case of the CFRP (Fig 11A). In this case, there is a point beyond which the addition of more material does not lead to further improvements in the seismic behaviour.

The results of the cost-benefit analysis have shown that the addition of CFRP bands has been the most expensive solution. Therefore, the implementation of this technique must be accurately analysed in order to reduce costs. Finite elements analysis should be carried out to determine the points of local failure of the walls, to optimize the use of the retrofitting material. On the other hand, the solution with best cost-benefit ratio has been the installation of encirclements in the openings. This technique is mainly focused on reinforcing local parts of the wall, which represent the major drawbacks of URM buildings. Thus, this technique can produce a considerable reduction of the seismic vulnerability of these buildings while avoiding elevated costs.

As a result, the most effective solutions have proved to be the addition of encirclements with L120.12 profiles and steel grids of Ø8 mm spaced 20 cm. Adding encirclements is less expensive but modifies the aspect of the building. Adding steel grids does not affect the aspect of the building but it is three times more expensive. In the context of the PERSISTAH project, the encirclements have been considered the most suitable retrofitting technique, provided the large amount of buildings to be retrofitted and the moderate seismicity of the region.

## Supporting information

S1 File DWG retrofitting details. (DWG)

S2 File DWG building configuration. (DWG)

S3 File RAR ENCIRCLEMENT L-120. This includes the Tremuri modelling and results files.
(RAR)

S4 File RAR GRID o8c20. This includes the Tremuri modelling and results files.
(RAR)

S5 File RAR CFRP 39-50E. This includes the Tremuri modelling and results files.
(RAR)
